# Ultrasonic-assisted extraction brings high-yield polysaccharides from *Kangxian* flowers with cosmetic potential

**DOI:** 10.1016/j.ultsonch.2023.106626

**Published:** 2023-09-30

**Authors:** Yang Zhang, Yihui Liu, Yingying Cai, Yuping Tian, Lianfa Xu, Aibei Zhang, Chen Zhang, Shushu Zhang

**Affiliations:** School of Biology and Food Engineering, Changshu Institute of Technology, Changshu 215500, Jiangsu, China

**Keywords:** Polysaccharides, *Kangxian* flowers, Ultrasonic-assisted extraction, Antioxidation, Moisture absorption, Moisture retention

## Abstract

The purpose of this work was to ameliorate the yield of polysaccharides from *Kangxian* flowers (PKFs) using ultrasonic-assisted extraction (UAE) and to explore the structural features and cosmetic potential of PKFs obtained under the optimized UAE. According to response surface methodology, the optimal UAE for PKFs were liquid-to-solid ratio of 59: 1 mL/g, ultrasonic power of 404 W, time of 48 min, and temperature of 66℃, under which the yield of PKFs reached 26.8 ± 1.76 %, 2.6-folds higher than hot-water extraction. By comparing the microstructures, it can be seen that ultrasonication exerted great damage on *Kangxian* flowers to promote the outflow of PKFs. Various analyses revealed that the PKFs extracted with UAE were acidic polysaccharides with relatively lower molecular weights of 0.92 – 76.9 kDa, negatively charged potential of −16.3 mV, and arabinose the dominant monosaccharide followed by galactose. These structural features conferred PKFs pronounced antioxidant, moisture absorption and retention properties, showing great potential for cosmetic application.

## Highlights

• Polysaccharides from *Kangxian* flowers (PKFs) were first extracted with ultrasound.
• Ultrasonication promoted the outflow of PKFs via greatly damaging materials.
• PKFs extracted with ultrasound were acidic low-molecular-weight polysaccharides.
• PKFs extracted with ultrasound elicited great potential for cosmetic application.

## 1 Introduction

Polysaccharides that usually comprise more than 10 monosaccharides, are considered to be the most important macromolecules in organisms [1]. Numerous studies have confirmed that polysaccharides own a wide range of health-benefiting properties, coupled with favorable hydrophilicity, safety and biodegradability, polysaccharides are popularly applied in foods, pharmaceuticals and other industrial fields [2]. In the cosmetic industry, polysaccharides equally serve as sustainable natural resources due to pronounced cosmetic functions, in particular of moisture-preserving and antioxidant capacities. As being essential for antiaging care, moisture retention keeps the skin soft and elastic, while antioxidation delays the skin senescence via scavenging free radicals [3], [4]. The moisture retention and antioxidation of polysaccharides mainly rely on their multiple polar groups, especially hydroxyl and carboxyl groups, which not only bind water through hydrogen bond association, but also provide hydrogens to quench free radicals [5], [6].

Traditional extraction techniques such as hot-water extraction (HWE) suffer from disadvantages of low yield and low efficiency, which greatly limit the industrial development and application of polysaccharides [7]. In order to overcome the drawbacks, modern auxiliary techniques, including ultrasonic-assisted extraction (UAE), pulsed electric field-assisted extraction (PEFAE), enzyme-assisted extraction (EAE) and microwave-assisted extraction (MAE), etc. have been successfully developed for the efficient extraction of polysaccharides in the past few decades [8]. In comparison, UAE is a non-thermal extraction technology with the advantages of lower energy consumption and shorter processing time. UAE utilizes the cavitation effect to promote the release and diffusion of polysaccharides from plant materials, bringing a highly efficient extraction outcome [9]. Besides, proper ultrasonication can optimize the physiochemical properties even bioactivities of extractable polysaccharides [10]. For example, the yield of polysaccharides from *Dictyophora indusiata* extracted with UAE was higher than those of HWE and MAE [11]; In addition to yield, UAE increased the uronic acid content, total flavonoid content, water solubility, etc. of polysaccharides from *Panax notoginseng* flower as compared with HWE [12]; More importantly, ultrasonication improved the antioxidant capacities of polysaccharides from *Ganoderma lucidum* [13], *Dendrobium officinale* [14], *Auricularia auricula* [15], and so on.

About 600 species of the genus *Dianthus* have been found in the world, many of which are cultivated as cut flowers or garden flowers [16]. Carnation, also known as *Dianthus caryophyllus* is of great economic value. It is the most popular member of genus *Dianthus* and the second most common cut flowers worldwide [17]. *Kangxian* flower, an edible species of carnation is mainly distributed in high-altitude areas, especially in the Tibetan Plateau being deemed as “Tibetan Holy Flower”. In China, the edible history of *Kangxian* flower can be traced back to hundreds of years ago. The Compendium of Materia Medica described that it has sweet taste and slight coolness as well as benefits liver and kidney [18]. According to the ethnomedicinal uses, various *Kangxian* flower-based tonic teas have been available on the market and exported abroad due to the fast development of international logistics [19], [20], [21]. At present, *Kangxian* flower-related products are still limited to tonic teas, lacking high-value and comprehensive development. Thus, the bioactive constituents present in *Kangxian* flower urgently need to be explored.

In this work, the UAE process was first optimized to extract polysaccharides from *Kangxian* flowers (PKFs). After purification and characterization, the cosmetic potential of PKFs, including antioxidant, moisture absorption and retention properties were then evaluated. The present contribution will evidence the value-added utilization of *Kangxian* flowers, more importantly further enrich the research cases on UAE of cosmetic polysaccharides.

## 2 Materials and methods

### 2.1 Materials and reagents

Dried *Kangxian* flowers were bought from Bozhou Chinese Herbal Medicine Market (Bozhou, Anhui, China) on 20 October 2022, and authenticated by Dr. Zhaowei Yan from Soochow University (Suzhou, Jiangsu, China). The involved reagents and drugs were of analytical grade and provided by local suppliers.

### 2.2 Ultrasonic-assisted extraction (UAE) of polysaccharides from Kangxian flowers (PKFs)

Dried *Kangxian* flowers were cut into pieces and passed through a 40-mesh sieve. After being soaked in water for 4 h at various liquid-to-solid ratios (20: 1 – 60: 1 mL/g), the mixture suspension was placed in a XH-2008D ultrasonic extractor (Xianghu Technology, Beijing, China) and extracted at different powers (200 – 600 W) and temperatures (30 – 70℃) for 10 – 50 min. After filtration under vacuum, the filtrate was metered to calculate extraction yield, and then condensed to 1/4 of the original volume, followed by adding 3-fold anhydrous ethanol to precipitate crude PKFs at 4℃, which were collected by centrifugation and dried by lyophilization [22].

Total carbohydrates were determined using a standard glucose curve of *Y* = 0.012*X* + 0.0224 (R^2^ = 0.9997) based on the principle of phenol–sulfuric acid method [23].

Extraction yield of crude PKFs was calculated according to Eq. (1).

(1) $$Yield\left(\%\right)=\left[\left(C\times V\times d\right)/m\right]\times 100$$

where C-content of total carbohydrates (mg/mL), V-original volume of filtrate (mL), d-dilution ratio, and m-weight of *Kangxian* flower powders (g).

### 2.3 Single factor test

The four UAE parameters, including liquid-to-solid ratio (A, 20: 1 – 60: 1 mL/g), ultrasonic power (B, 200 – 600 W), ultrasonic time (C, 10 – 50 min), and temperature (D, 30 – 70℃) were appraised via single factor test using PKFs yield as the evaluation index.

### 2.4 Response surface optimization

Design-Expert 13 software was used to design and analyze the four-factor (A – D) and three-level (-1 – 1) response surface methodology (RSM) according to Box-Behnken design (BBD). The proper values of A – D were selected based on the results of single factor test using PKFs yield as the response.

### 2.5 Comparison experiment

According to the optimized UAE condition and previous literature [9], a comparison experiment was designed to explore the contribution of ultrasonication to PKFs yield. In brief, a hot-water extraction (HWE) was performed by fixing the liquid-to-solid ratio at 59: 1 mL/g and changing the temperature into 80℃ and the time into 4 h. The PKFs yield and microstructure of extraction residual obtained under HWE were compared with those of optimized UAE.

### 2.6 Purification of crude PKFs

Free proteins in crude PKFs were removed by the Sevag method for three times [24]. Then, the deproteinized PKFs were further purified with diethylaminoethyl (DEAE) cellulose column chromatography using 0.5 M NaCl as the mobile phase. The subsequent eluent was dialyzed for three times and lyophilized to produce the purified PKFs [25].

### 2.7 Basic characterization of purified PKFs

#### 2.7.1 General components

Total carbohydrates were measured according to the method described in Section 2.2. Other components, including proteins, uronic acids and sulfates were determined by the Bradford method [26] with a standard bovine serum albumin curve of *Y* = 7.25*X* − 0.0356 (R^2^ = 0.9998), the *m*-hydroxybiphenyl method [27] with a standard galacturonic acid curve of *Y* = 8.4688*X* − 0.0187 (R^2^ = 0.9992), and the barium chloride-gelatin method [28] with a standard potassium sulfate of *Y* = 8.4688*X* − 0.0187 (R^2^ = 0.9995), respectively.

#### 2.7.2 Monosaccharide composition and molecular weight

Trifluoroacetic acid was used to hydrolyze purified PKFs into monosaccharides, which were analyzed by a Dionex^TM^ ICS-5000 ion chromatograph (Thermo Fisher, Waltham, MA, USA) equipped with a pulsed ampere detector and a CarboPac PA20 ion chromatographic column. The injection volume was 20 µL, and velocity of flow was 0.5 mL/min with the following gradient elution: 0 – 21 min, 98 % ultrapure water + 2 % 250 mM NaOH solution; 21.1 – 30 min, 93 % ultrapure water + 2 % 250 mM NaOH solution + 5 % 1 M NaAc solution [29].

The molecular weight (Mw) of purified PKFs was determined by high performance size exclusion chromatography (HPSEC) using a TSKgel^TM^ G4000PWXL column. A 1220 Infinity II high performance liquid chromatograph (Agilent, Palo Alto, CA, USA) served as the analyzer. The injection volume was 20 µL, mobile phase was ultrapure water with a flow rate of 0.9 mL/min, and the column temperature was 45℃ [25].

#### 2.7.3 Particle size and zeta potential

The particle size and zeta potential of purified PKFs were measured with a Malvern^TM^ ZS90 Zetasizer Nano (Malvern Panalytical, Malvern, Worcestershire, UK) at a concentration of 1 mg/mL in distilled water.

#### 2.7.4 Ultraviolet (UV) and infrared (IR) analysis

The UV spectrum of purified PKFs within 200 – 800 nm was scanned using a Lambda 750 ultraviolet spectrophotometer (PerkinElmer, Waltham, MA, USA), and IR spectrum within 4000 – 400 cm^−1^ was scanned using a Spotlight 200i Fourier transform infrared microscope (PerkinElmer, Waltham, MA, USA).

#### 2.7.5 Scanning electron microscopy (SEM)

The untreated *Kangxian* flower powders, dried residuals after extraction, and purified PKFs were placed onto silicon pellets, respectively. After being spattered with gold powders, a Regulus 8100 ultrahigh-resolution scanning electron microscope (Hitachi, Tokyo, Japan) served to exhibit the SEM images.

#### 2.7.6 Atomic force microscope (AFM)

Ten microliters of purified PKFs at a concentration of 1 mg/mL in ultrapure water were loaded onto a cleaved mica sheet. After being dried under vacuum, a XE-120 atomic force microscope (Park Systems, Suwon, South Korea) was applied to exhibit the AFM image.

### 2.8 Antioxidant activity

#### 2.8.1 Hydroxyl radical-scavenging test

One milliliter of purified PKFs solution (0.2 – 1 mg/mL) was sequentially mixed with 1 mL of 6 mM FeSO_4_ solution, 1 mL of 6 mM salicylic acid in ethanol, and 1 mL of 6 mM H_2_O_2_ solution. After incubation at 37℃ for 30 min, the absorbance at 510 nm (As) was read [22]. Distilled water replacing PKFs served as the blank control (A_0_), and the reaction system lacking H_2_O_2_ was applied as the normal control (Ac). *L*-Ascorbic acid (LAA) served as the positive control. The scavenging rate against hydroxyl radical was calculated based on Eq. (2).

(2) $$Scavenging\;rate\;against\;hydroxyl\;radical\left(\%\right)=\left[\left(\mathrm{As}-A_{0}\right)/\left(\mathrm{Ac}-A_{0}\right)\right]\times 100$$

#### 2.8.2 1, 1-Diphenyl-2-picrylhydrazyl (DPPH) radical-scavenging test

One milliliter of purified PKFs solution (0.1 – 0.8 mg/mL) was blended with 1 mL of 0.1 mM DPPH in ethanol and reacted under the dark for 30 min, then the absorbance at 517 nm (As) was determined. Distilled water replacing PKFs served as the blank control (A_0_), and the reaction system without DPPH was used as the normal control (Ac). LAA acted as the positive control [25]. The scavenging rate against DPPH radical was reckoned according to Eq. (3).

(3) $$Scavenging\;rate\;against\;DPPH\;radical\left(\%\right)=\left[\left(\mathrm{As}-Ac\right)/A_{0}\right]\times 100$$

### 2.9 Moisture absorption and retention

#### 2.9.1 Moisture-absorbing property

All the samples (purified PKFs, chitosan and glycerol) were dried at 100℃ for 4 h, then 0.5 g of each sample (W_0_) was placed in a humidity chamber with saturated (NH_4_)_2_SO_4_ (81 % RH) at room temperature. At indicated time (4 – 48 h), the samples were respectively weighed (Wt) [6]. The moisture-absorbing rate (Ra) was calculated based on Eq. (4).

(4) $$Ra\left(\%\right)=\left[\left(Wt-W_{0}\right)/W_{0}\right]\times 100$$

#### 2.9.2 Moisture-preserving capacity

Fifty milligrams of each sample (W_0_) were placed in a humidity chamber with distilled water. After being humidified for 24 h, the samples were weighed (Wm) and transferred to desiccators with dried silica gel to dehydrate at room temperature. At indicated time (4 – 48 h), the samples were respectively weighed again (Wt) [30]. The moisture-preserving rate (Rp) was estimated according to Eq. (5).

(5) $$Rp\left(\%\right)=\left[\left(Wm-Wt\right)/\left(\mathrm{Wm}-W_{0}\right)\right]\times 100$$

### 2.10 Statistical analysis

The results of experiments in triplicate were presented as means ± SD. The ANOVA or *t*-test were performed to analyze the statistical differences between data using a GraphPad Prism software 9.5.1, and *P* < 0.05 was deemed as the lowest level of statistical difference.

## 3 Results and discussion

### 3.1 Effects of UAE parameters on PKFs yield

**Fig. 1:**
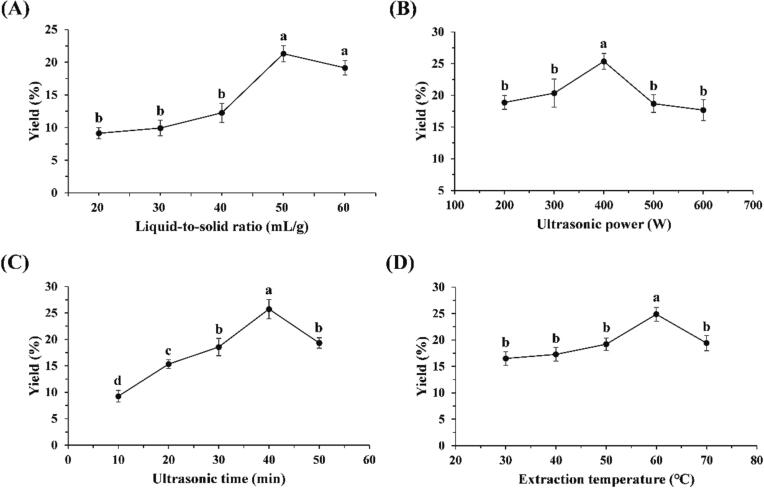
The effects of liquid-to-solid ratio (**A**), ultrasonic power (**B**), ultrasonic time (**C**), and extraction temperature (**D**) on PKFs yield. Different lowercase letters represented statistical differences.

As shown in Fig. 1A, PKFs yield increased as liquid-to-solid ratio being elevated from 20: 1 to 50: 1 mL/g, and decreased when liquid-to-solid ratio exceeded 50: 1 mL/g. Excess water makes the raw material overswelling, which may cause more extractable PKFs to be adsorbed on the surface, resulting in a lower yield [31]. As exhibited in Fig. 1B, with increasing ultrasonic power from 200 to 400 W, PKFs yield was gradually improved, especially from 300 to 400 W, significant difference was found (*P* < 0.05), but continuous increase in ultrasonic power led to marked decrease in PKFs yield (*P* < 0.01). Mechanical force generated by ultrasound facilitates the release and diffusion of PKFs, but too strong ultrasonication can degrade polysaccharides, leading to a reduction in yield [22]. Similarly, as presented in Fig. 1C, too long ultrasonication can also destroy PKFs, resulting in a decrease in yield [7]. PKFs yield significantly increased as ultrasonic time being prolonged from 10 to 40 min (*P* < 0.05 or *P* < 0.01), and notably deceased when ultrasonic time reached 50 min (*P* < 0.01). Fig. 1D displayed the influence of temperature on PKFs yield. It can be noted that with the elevation of temperature from 30 to 60℃, particularly from 50 to 60℃, PKFs yield markedly increased (*P* < 0.05). When temperature was raised to 70℃, PKFs yield significantly decreased (*P* < 0.05). Proper heating enhances the thermal diffusion of PKFs, but too high temperature as hot-water extraction can cause a reduction in yield via degrading structures [22].

Through single-factor experiment, the liquid-to-solid ratio (A) of 50: 1 mL/g, ultrasonic power (B) of 400 W, time (C) of 40 min and temperature (D) of 60℃ were selected for the experimental design of RSM-BBD optimization.

### 3.2 Response surface optimization on UAE conditions for PKFs extraction

Table 1 showed the design and results of response surface trial based on single-factor experiment, and Eq. (6) interpreted the regression relation between PKFs yield (*Y*) and coded factors (A – D).

(6) $$Y=24.22+2.86\times A+0.6\times B+0.3167\times C+1.59\times D+0.525\times AB+3.15\times AC+0.5\times AD-1.5\times BC-0.025\times BD+2.4\times CD-2.41\times A^{2}-3.22\times B^{2}-2.49\times C^{2}-3.11\times D^{2}$$

**Table 1:** Experimental design based on RSM-BBD and the results.

Run	Coded factors				*Y* (%)
	A (mL/g)	B (W)	C (min)	D (℃)	
1	0 (50)	0 (4 0 0)	0 (40)	0 (60)	23.4
2	−1 (40)	1 (5 0 0)	0 (40)	0 (60)	15.7
3	0 (50)	0 (4 0 0)	0 (40)	0 (60)	24.3
4	0 (50)	−1 (3 0 0)	−1 (30)	0 (60)	16.3
5	0 (50)	0 (4 0 0)	0 (40)	0 (60)	24.3
6	−1 (40)	−1 (3 0 0)	0 (40)	0 (60)	15.7
7	0 (50)	1 (5 0 0)	0 (40)	−1 (50)	16.8
8	0 (50)	0 (4 0 0)	−1 (30)	−1 (50)	18.8
9	−1 (40)	0 (4 0 0)	−1 (30)	0 (60)	19.5
10	0 (50)	0 (4 0 0)	1 (50)	−1 (50)	15.1
11	0 (50)	−1 (3 0 0)	1 (50)	0 (60)	19.7
12	0 (50)	0 (4 0 0)	−1 (30)	1 (70)	17.4
13	−1 (40)	0 (4 0 0)	0 (40)	−1 (50)	15.1
14	0 (50)	−1 (3 0 0)	0 (40)	−1 (50)	15.2
15	−1 (40)	0 (4 0 0)	0 (40)	1 (70)	16.5
16	0 (50)	1 (5 0 0)	−1 (30)	0 (60)	20.3
17	1 (60)	0 (4 0 0)	0 (40)	1 (70)	23.3
18	0 (50)	1 (5 0 0)	1 (50)	0 (60)	17.7
19	1 (60)	0 (4 0 0)	0 (40)	−1 (50)	19.9
20	0 (50)	0 (4 0 0)	0 (40)	0 (60)	24.6
21	1 (60)	−1 (3 0 0)	0 (40)	0 (60)	20.5
22	1 (60)	0 (4 0 0)	1 (50)	0 (60)	25.4
23	0 (50)	1 (5 0 0)	0 (40)	1 (70)	20.5
24	1 (60)	0 (4 0 0)	−1 (30)	0 (60)	18.7
25	0 (50)	−1 (3 0 0)	0 (40)	1 (70)	19.0
26	1 (60)	1 (5 0 0)	0 (40)	0 (60)	22.6
27	−1 (40)	0 (4 0 0)	1 (50)	0 (60)	13.6
28	0 (50)	0 (4 0 0)	1 (50)	1 (70)	23.3
29	0 (50)	0 (4 0 0)	0 (40)	0 (60)	24.5

**Fig. 2:**
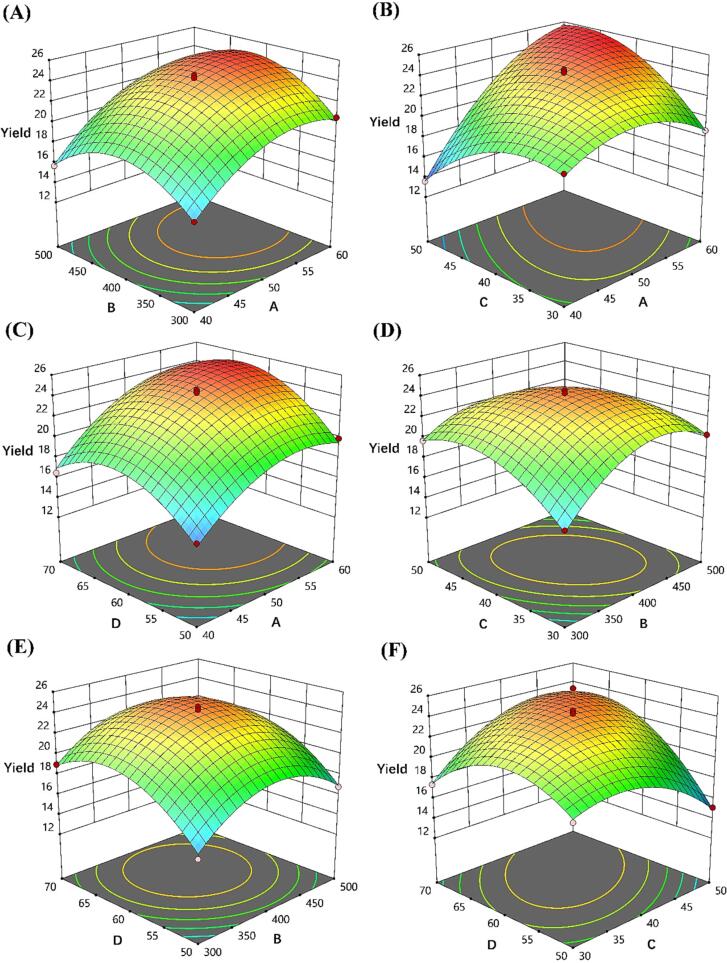
The 3D surface plots showing interactions of any two variables on PKFs yield.

As summarized in Table 2, the significance for Model (*P* < 0.01) and insignificance for Lack of Fit (*P* > 0.05) indicated that present model was reliable to forecast and analyze the UAE of PKFs [32]. Terms of A – D, AB, AC, AD, BC, CD, and A^2^ – D^2^ were remarkable (*P* < 0.05 or *P* < 0.01), suggesting that these items can significantly influence PKFs yield, and the influence ranked as A > D > B > C [33]. The Adj R^2^ was 0.9856, demonstrating that the model can interpret 98.65 % of the variations, and a relatively lower C.V. (2.15 %) verified that the results of actual experiments were credible [34]. Fig. 2 visualized the interactions of any two variables on PKFs yield. It can be observed that PKFs yield first increased with the increase of A – D, and then as A – D continued to increase, PKFs yield decreased, which was in line with single-factor experiment (Fig. 1). The surface plot with a steeper slope indicates that the interaction effect is more significant, i.e., the response is more sensitive to the changes of variables [35]. As can be seen by comparing the slope of surfaces, A outperformed other variables on PKFs yield, which was consistent with ANOVA results (Table 2).

**Table 2:** ANOVA for the regression model predicting PKFs extraction.

Source	Sum of Squares	df	Mean Square	*F*-value	*P*-value	Significance
Model	342.25	14	24.45	137.79	< 0.0001	^**^
A	98.04	1	98.04	552.6	< 0.0001	^**^
B	4.32	1	4.32	24.35	0.0002	^**^
C	1.2	1	1.2	6.78	0.0208	*
D	30.4	1	30.4	171.35	< 0.0001	^**^
AB	1.1	1	1.1	6.21	0.0258	*
AC	39.69	1	39.69	223.71	< 0.0001	^**^
AD	1.0	1	1.0	5.64	0.0324	*
BC	9.0	1	9.0	50.73	< 0.0001	^**^
BD	0.0025	1	0.0025	0.0141	0.9072	
CD	23.04	1	23.04	129.86	< 0.0001	^**^
A^2^	37.54	1	37.54	211.61	< 0.0001	^**^
B^2^	67.18	1	67.18	378.68	< 0.0001	^**^
C^2^	40.32	1	40.32	227.29	< 0.0001	^**^
D^2^	62.57	1	62.57	352.67	< 0.0001	^**^
Residual	2.48	14	0.1774			
Lack of Fit	1.58	10	0.1576	0.6942	0.7093	
Pure Error	0.908	4	0.227			
Cor Total	344.73	28				
R^2^	0.9928	Adj R^2^	0.9856	C.V.	2.15 %	

*P < 0.05 and ^**^P < 0.01.

The model-predicted conditions for PKFs extraction were: liquid-to-solid ratio (59.3706: 1 mL/g), ultrasonic power (403.798 W), time (48.2009 min), and temperature (66.1443℃), under which the predicted yield of PKFs was 27.0816 %. To facilitate operation, the predicted conditions were adjusted to be liquid-to-solid ratio (59: 1 mL/g), ultrasonic power (404 W), time (48 min), and temperature (66℃), under which the practical yield of PKFs was 26.8 ± 1.76 %, with an error of −1.04 % in comparison with model prediction, further confirming the feasibility and accuracy of this model in predicting the UAE of PKFs.

### 3.3 Comparison experiment

**Fig. 3:**
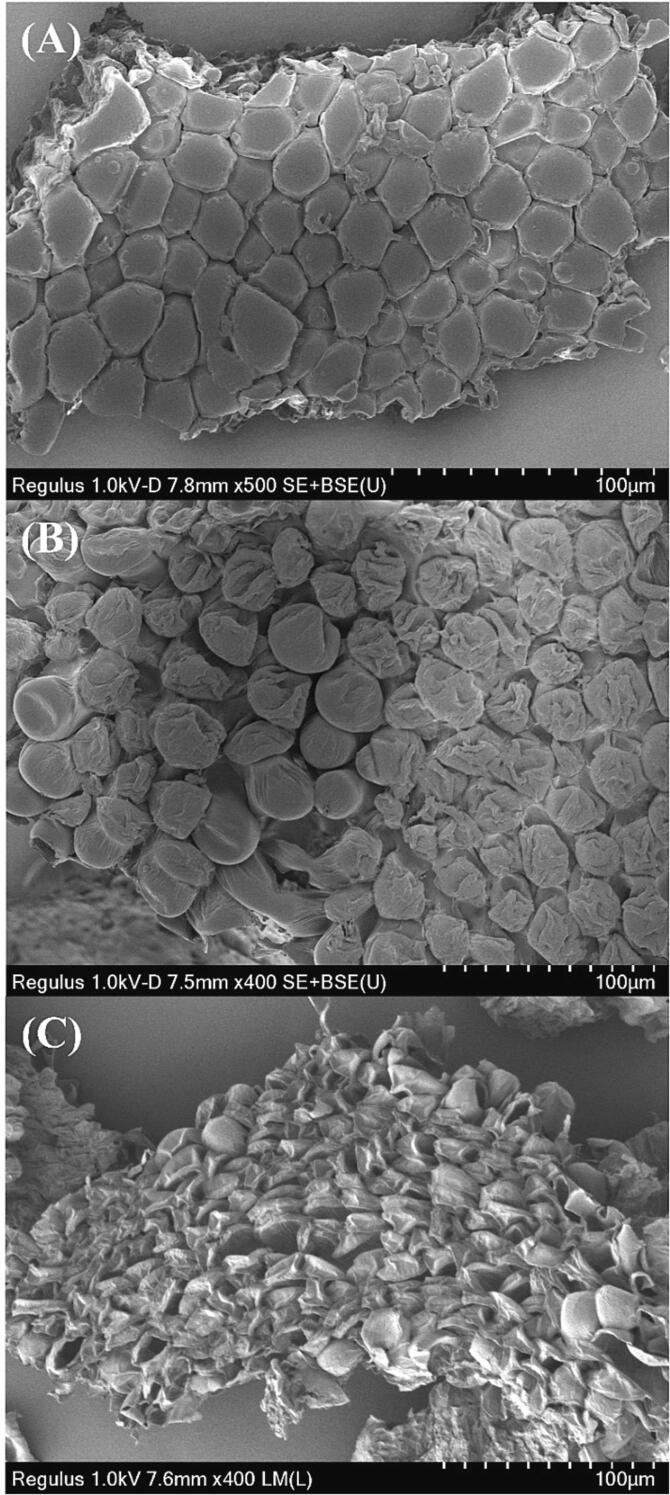
The SEM observations of untreated *Kangxian* flowers (**A**), HWE-treated material (**B**) and UAE-treated material (**C**).

To explore the contribution of ultrasonication to PKFs yield, a HWE of PKFs was conducted and compared with UAE. The PKFs yield with HWE was 10.4 ± 1.41 %, markedly lower (*P* < 0.01) than that of UAE (26.8 ± 1.76 %). By comparing the SEM images of untreated and treated *Kangxian* flowers (Fig. 3), it can be observed that the microstructure of untreated material tightly packed rhombus-shaped lumps (Fig. 3A), after HWE treatment, most of lumps began to shrink (Fig. 3B). The microstructure of UAE-treated material appeared obvious destruction and the lumps entirely shriveled after 48 min-ultrasonication (Fig. 3C). These results showed that ultrasonication exerted great damage on the microstructure of *Kangxian* flowers to promote the outflow of PKFs, thereby improving the yield significantly.

### 3.4 Basic characterization

**Fig. 4:**
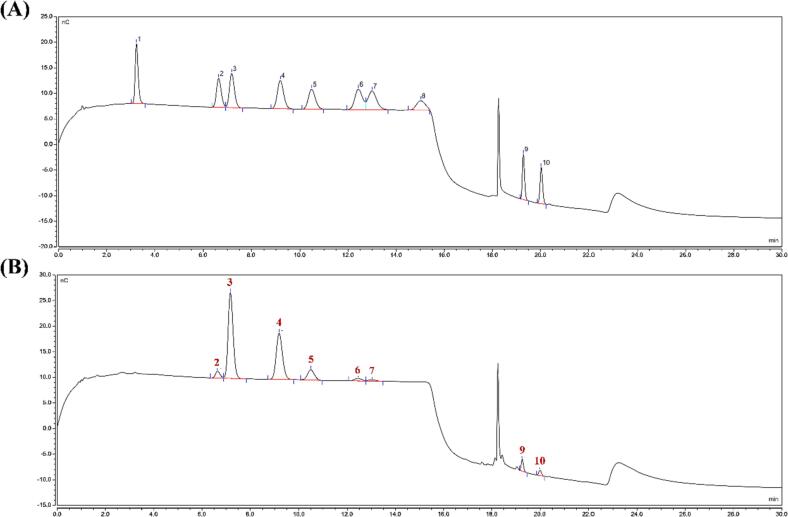
The ion chromatograms for sugar standards (**A**) and monosaccharides of PKFs (**B**). Peaks in chromatograms were 1-fucose, 2-rhamnose, 3-arabinose, 4-galactose, 5-glucose, 6-xylose, 7-mannose, 8-fructose, 9-galacturonic acid, and 10-glucuronic acid.

Purified PKFs comprised 70.7 ± 1.26 % carbohydrates, 22.9 ± 1.11 % uronic acids and 12.8 ± 0.61 % proteins. Fig. 4 exhibited the monosaccharide composition, as compared with sugar standards (Fig. 4A), PKFs were composed of rhamnose, arabinose, galactose, glucose, xylose, mannose, galacturonic acid, and glucuronic acid at molar ratios of 3.3: 53.3: 30.2: 7.3: 2.3: 1.0: 2.6: 1.3 (Fig. 4B), suggesting that PKFs were heteropolysaccharides with arabinose the dominant monosaccharide followed by galactose.

**Fig. 5:**
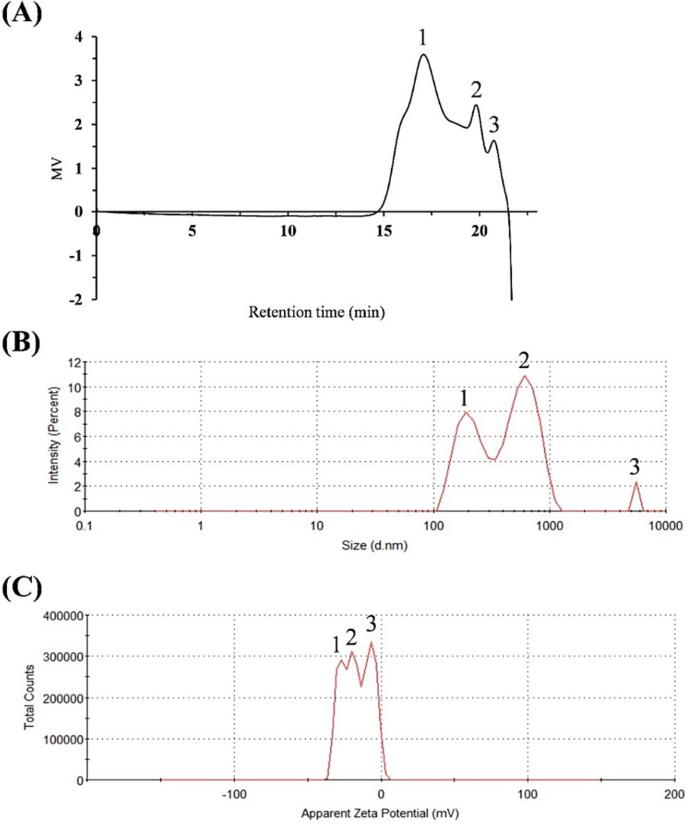
The HPSEC chromatogram (**A**), particle size distribution (**B**) and zeta potential map (**C**) of PKFs.

From the HPSEC chromatogram (Fig. 5A), it could be concluded that PKFs contained three polysaccharide fractions, corresponding to peak 1 with Mw of 76.9 kDa (73.24 %), peak 2 (2.88 kDa, 17.89 %), and peak 3 (0.92 kDa, 8.87 %), which was further confirmed by particle size and zeta potential. The particle size distribution map appeared three peaks (Fig. 5B), corresponding to peak 1 (217.3 nm, 40.6 %), peak 2 (611 nm, 57.2 %), and peak 3 (5560 nm, 2.2 %), with average particle size of 547.7 nm. The zeta potential map also appeared three peaks (Fig. 5C), corresponding to peak 1 (-27.4 mV, 28.6 %), peak 2 (-18.6 mV, 33 %), and peak 3 (-7.14 mV, 38.4 %), with average zeta potential of −16.3 mV. The negatively charged property of PKFs could be principally contributed by uronic acids [36], which accounted for 22.9 ± 1.11 % in purified PKFs. The particle size distribution of PKFs was broad and uneven, which may be due to the fact that PKFs belonged to heteropolysaccharides consisting of eight monosaccharides (Fig. 4B) [37]. This phenomenon was also observed in other investigations, suggesting that PKFs could aggregate in water to some extent [38], [39]. As for zeta potential, the value > 30 mV or < -30 mV is deemed stable [40]. The average zeta potential of PKFs was −16.3 mV, higher than −30 mV, indicating that PKFs may be unstable in water being consistent with the influence of particle size.

**Fig. 6:**
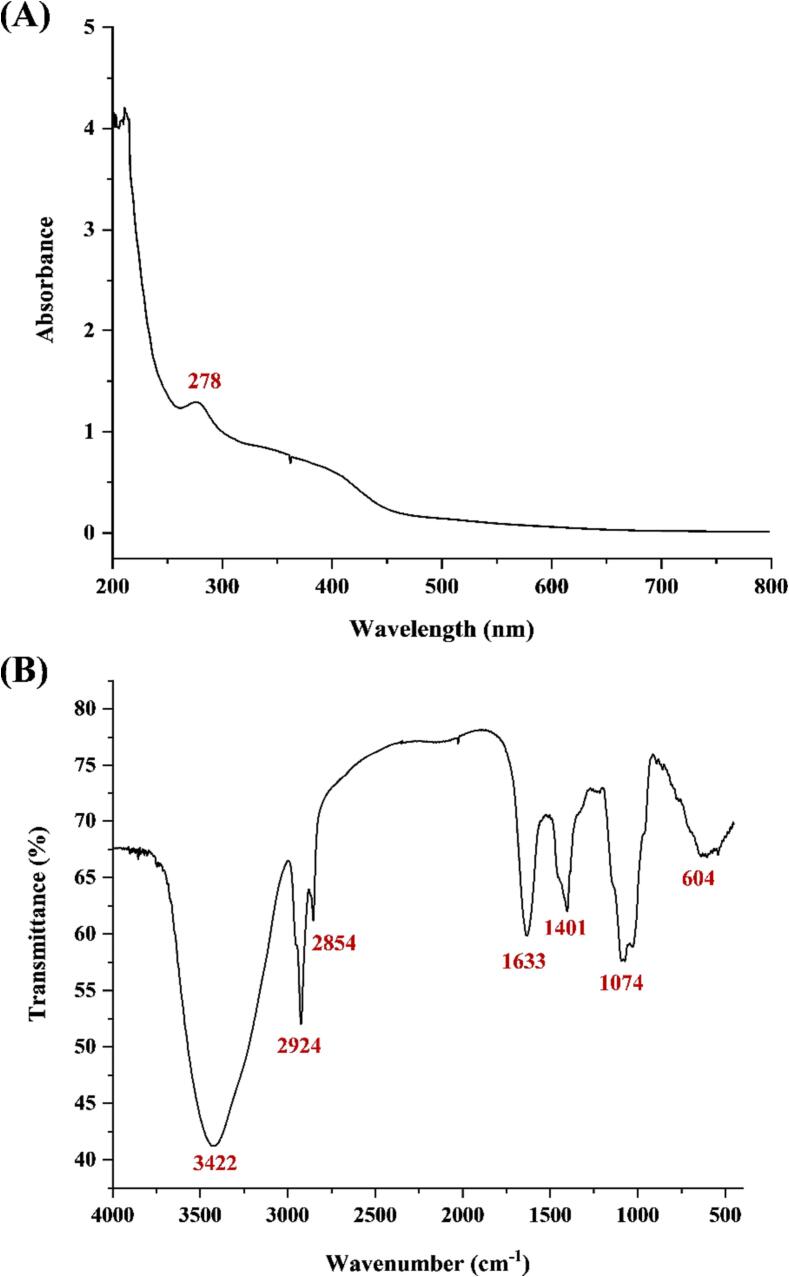
The UV (**A**) and IR (**B**) spectra of PKFs.

The UV spectrum of purified PKFs showed a characteristic peak for proteins at around 280 nm (Fig. 6A), which was suspected to predominantly binding proteins, since free proteins had been basically cleaned by Sevag reagent [41]. Fig. 6B exhibited the IR spectrum. The broad peak at 3422 cm^−1^ referred to the stretching vibration of –OH, peaks at 2924 and 2854 cm^−1^ were from the stretching and bending vibrations of –CH, and peak at 1633 cm^−1^ was assigned to the stretching vibration of -C = O [42]. The peak at 1401 cm^−1^ further confirmed the presence of uronic acids [43], peak at 1074 cm^−1^ accorded with the stretching vibration of C-O-C [44], and peak at 604 cm^−1^ was the result of pyranose ring [45]. The FT-IR characteristic absorption peaks proved that PKFs belonged to polysaccharides, especially the acidic polysaccharides.

**Fig. 7:**
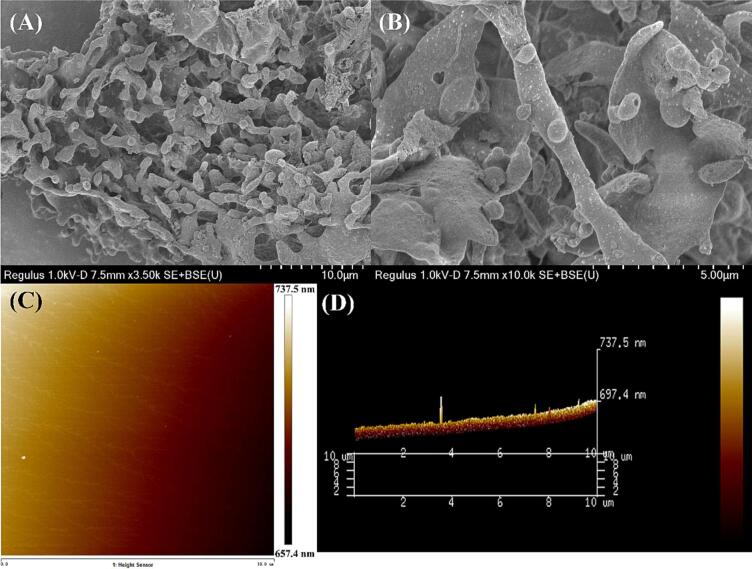
The SEM images (**A** – **B**), 2D (**C**) and 3D (**D**) AFM images of PKFs.

The morphology structure of purified PKFs was visualized by SEM under magnifications of 3,500 and 10,000× (Fig. 7 A – B). It can be seen that the surface of PKFs exhibited rough, porous, and irregular coral-like appearance, which was similar to previous findings that could be caused by the crosslinks and aggregations between sugar chains [46]. Fig. 7C – D displayed the 2D and 3D AFM images of purified PKFs on the nanometer scale. Under AFM, the morphology of PKFs appeared conical lumps (Fig. 7D), and the heights of these lumps ranged from 657.4 to 737.5 nm (Fig. 7C). In general, the height of a single polysaccharide chain spans from 0.1 to 1.0 nm [47], indicating that intensive aggregations had been occurred in PKFs.

### 3.5 Antioxidant activity

Hydroxyl radical is the most reactive member of common reactive oxygen species (ROS). It is widely distributed in the body and can react with almost all the biomolecules. Thus, hydroxyl radical is one of the main causes of senescence and various diseases [48]. On the contrary, DPPH radical is one of the reactive nitrogen species (RNS) with stable property, and only the antioxidant with higher activity can effectively scavenge it [49].

**Fig. 8:**
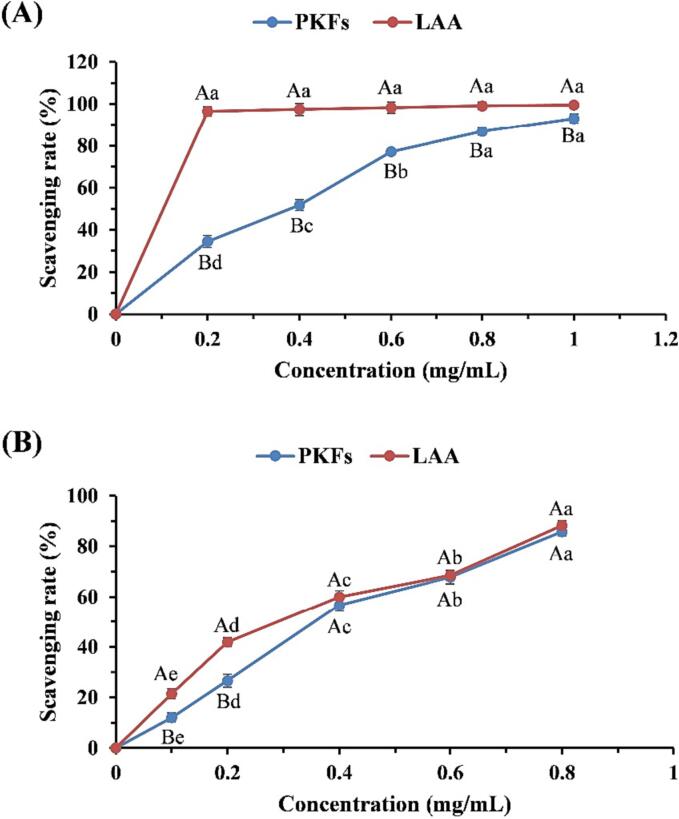
The scavenging capacities of PKFs against hydroxyl (**A**) and DPPH (**B**) radicals. Different capital letters indicated statistical differences between groups at the same concentration, and different lowercase letters represented statistical differences within group.

As shown in Fig. 8A, within 0.2 – 1 mg/mL, PKFs scavenged hydroxyl radical in a concentration-dependent manner (*P* < 0.01). Despite continuous increase in concentration led to a higher scavenging rate at 1 mg/L (92.9 ± 2.14 %), when compared with that of 0.8 mg/L (86.8 ± 1.69 %), there was no statistical difference (*P* > 0.05). As exhibited in Fig. 8B, the DPPH radical-scavenging capacity of PKFs increased within 0.1 – 0.8 mg/mL in a concentration-dependent manner (*P* < 0.01). The scavenging rate maximized at 0.8 mg/mL was 85.8 ± 1.65 %, slightly lower that of LAA (88.2 ± 1.81 %), but remarkable difference was not observed (*P* > 0.05).

LAA is recognized as one of the most potent antioxidants, and few substances can surpass it. Not surprisingly, the scavenging rates of PKFs against hydroxyl radical were notably lower than those of LAA at any of the tested concentrations (*P* < 0.05 or *P* < 0.01). However, within 0.4 – 0.8 mg/mL, the scavenging rates of PKFs against DPPH radical were similar to those of LAA (*P* > 0.05), suggesting that PKFs possessed DPPH radical-scavenging capacity close to LAA.

Various structural parameters, such as monosaccharides, Mw and the presence of uronic acids determine the antioxidation of PKFs, and the activity is not the result of one polysaccharide fraction but the synergetic effect of multiple components [50]. In the near future, the activity-directed fractionation and in-depth characterization are needed to reveal the antioxidant structure–activity relationship and mechanism of PKFs.

### 3.6 Moisture absorption and moisture preservation

**Fig. 9:**
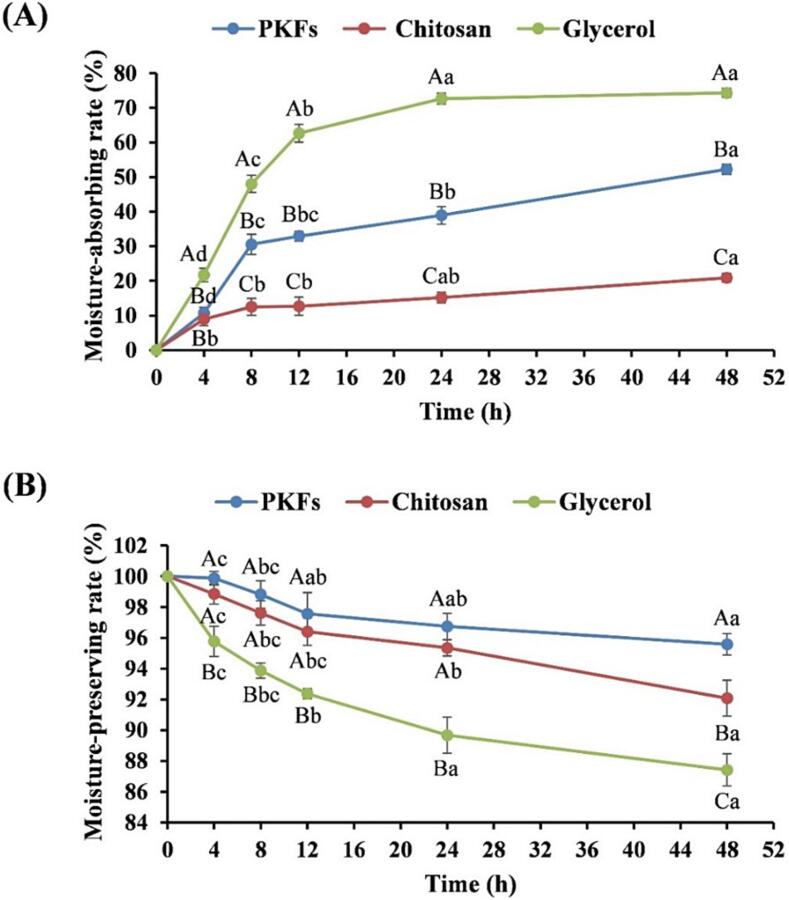
The moisture absorption (**A**) and moisture preservation (**B**) of PKFs. Different capital letters indicated statistical differences between groups at the same time, and different lowercase letters represented statistical differences within group.

The moisture-absorbing properties of PKFs and two controls (glycerol and chitosan) were showed in Fig. 9A. Under 81 % RH, PKFs absorbed moisture in the air in a time-dependent manner (*P* < 0.01). At 48 h, the moisture-absorbing rate reached 52.2 ± 1.41 %, which was significantly higher (*P* < 0.01) than that of chitosan (20.9 ± 1.41 %), but markedly lower (*P* < 0.01) than that of glycerol (74.3 ± 1.27 %). The results indicated that PKFs outperformed chitosan but underperformed glycerol on moisture absorption.

The moisture-preserving profiles were displayed in Fig. 9B. In a dry atmosphere, the fully humidified PKFs exerted excellent moisture-preserving capacity, especially within 12 – 48 h, significant difference in moisture-preserving rate was not noted (*P* > 0.05). The moisture-preserving rate of PKFs at 48 h was 95.6 ± 0.69 %, which was significantly higher (*P* < 0.05 or *P* < 0.01) than those of chitosan (92.1 ± 1.16 %) and glycerol (87.4 ± 1.04 %). The results suggested that PKFs outperformed both chitosan and glycerol on moisture preservation.

PKFs exhibited moderate moisture-absorbing property and excellent moisture-preserving performance. They could be preferentially contributed by the polar groups in PKFs, such as hydroxyl, carbonyl and carboxyl groups, which can bind water by hydrogen bond association [51]. In addition, the interweaving of polysaccharide chains with lattice can also ensure the water holding capacity of PKFs [6].

The first concern in cosmetic development is the moisturizing properties of the products, since adequate moisture can make the skin soft and elastic [3]. PKFs possessed excellent moisture-preserving performance, and the moisturizing rate was still > 90 % after 48 h in dry atmosphere, significantly higher than that of glycerol, a well-known moisturizer. This indicated that PKFs can prevent skin moisture loss even in dry environment and better meet the daily moisturizing needs of potential consumers. In addition, the antioxidant capabilities of the products are also crucial. The skin contains endogenous antioxidants such as glutathione, superoxide dismutase, and catalase, which can alleviate the skin damages caused by free radicals. However, aging and environmental factors can gradually decrease the endogenous antioxidants. It is therefore important to supplement the exogenous antioxidants for delaying the skin senescence via scavenging free radicals [4], [52]. PKFs exerted pronounced scavenging capacities against hydroxyl and DPPH radicals, especially for DPPH radical, with activity comparable to that of LAA, a renowned antioxidant. These results implied that PKFs possessed great potential to be used as antiaging ingredient for cosmetic products. Other cosmetic functions of PKFs, including whitening and freckle-removing properties deserve to be explored in the future. With regard to the possible instability of PKFs in aqueous solution disclosed by particle size and zeta potential, which hinted that the cosmetic development of PKFs should focus on emulsions and creams, and solution agents should be avoided as much as possible.

## 4 Conclusion

In the present investigation, the UAE process for PKFs was optimized by RSM-BBD. Under an optimized UAE condition (liquid-to-solid ratio of 59: 1 mL/g, ultrasonic power of 404 W at 66℃ for 48 min), the yield of PKFs reached 26.8 ± 1.76 %, significantly higher (*P* < 0.01) than HWE (10.4 ± 1.41 %). SEM observation further revealed that ultrasonication greatly damaged the microstructure of materials to facilitate the release of PKFs. The PKFs extracted with UAE were acidic polysaccharides with a negatively charged potential of −16.3 mV, a molecular weight range of 0.92 – 76.9 kDa, and a particle size of 547.7 nm. Meanwhile, PKFs were composed of eight monosaccharides with arabinose the dominant one followed by galactose. PKFs exhibited pronounced antioxidant, moisture absorption and retention properties, especially for DPPH radical-scavenging capacity and moisture-preserving performance. In summary, ultrasonic processing efficiently led to the PKFs with great cosmetic potential.

## Funding

The present investigation was supported by Suzhou Science and Technology Bureau (Grant No. SNG2022060), and 2023 Innovation Training Programs for College Students in Jiangsu Province.

## CRediT authorship contribution statement

**Yang Zhang:** Conceptualization, Methodology, Writing – review & editing, Funding acquisition, Project administration, Supervision. **Yihui Liu:** Conceptualization, Methodology, Investigation, Data curation, Writing – original draft, Funding acquisition. **Yingying Cai:** Methodology, Investigation, Data curation, Formal analysis. **Yuping Tian:** Resources, Formal analysis, Validation. **Lianfa Xu:** Investigation, Data curation. **Aibei Zhang:** Software, Visualization. **Chen Zhang:** Validation, Formal analysis. **Shushu Zhang:** Investigation.

## Declaration of Competing Interest

The authors declare that they have no known competing financial interests or personal relationships that could have appeared to influence the work reported in this paper.
